# Effect of foveal morphology on visual acuity in 4–6-year-old children with retinopathy of prematurity: a J-CREST study

**DOI:** 10.1038/s41598-022-20956-4

**Published:** 2022-09-30

**Authors:** Tomo Nishi, Tetsuo Ueda, Yuutaro Mizusawa, Kayo Shinomiya, Yoshinori Mitamura, Naoki Kimura, Fumi Gomi, Akiko Miki, Makoto Nakamura, Takamasa Kinoshita, Shumpei Obata, Masahito Ohji, Takuya Tsuji, Shigeo Yoshida, Nahoko Ogata

**Affiliations:** 1 Department of Ophthalmology, Nara Medical University, 840 Kashihara City, Nara, 634-8522 Japan; 2 Department of Ophthalmology, Tokushima University, Tokushima, Japan; 3 Department of Ophthalmology, Hyogo College of Medicine, Nishinomiya, Japan; 4 Division of Ophthalmology, Department of Surgery, Kobe University School of Medicine, Kobe, Japan; 5 Department of Ophthalmology, Sapporo City General Hospital, Sapporo, Japan; 6 Department of Ophthalmology, Shiga University of Medical Science, Otsu, Japan; 7 Department of Ophthalmology, Kurume University, Kurume, Japan

**Keywords:** Retinal diseases, Neonatology

## Abstract

Laser therapy is the most effective treatment considered for retinopathy of prematurity (ROP). We compared the foveal morphology of the retina in eyes with a history of ROP to that of full-term children. This cross-sectional comparative study included 74 patients with a history of ROP, aged 4–6 years. Among them, 41 underwent laser treatment for ROP. The clinical findings and retinal morphology in these patients were compared to that of 33 patients who had spontaneous ROP regression and 30 age-matched full-term controls. All the patients with ROP had 20/40 or better best-corrected visual acuity (BCVA). The foveal thickness was significantly thicker in laser-treated ROP eyes than in regressed ROP eyes and controls. The outer nuclear layer was significantly thicker, and the inner segment (IS) of the photoreceptors and the inner retinal layer were significantly thicker in the laser-treated ROP eyes than that in the control eyes. In the patients with ROP and controls, better BCVA was associated positively with deeper foveal depression, which was associated with a later gestational age. Our results suggest that prematurity and laser treatment affect the foveal morphology and BCVA.

## Introduction

Retinopathy of prematurity (ROP), which warrants treatment, has increased with the development of perinatal care^1^. ROP has a higher incidence of myopia, amblyopia, retinal detachment, strabismus, cataracts, glaucoma, and macular folds^1–5^. Angiogenesis in the retina begins from the posterior pole at 24–28 weeks of gestational age, and the peripheral retina is finally vascularized at 40 weeks^6^. Thus, retinal development, including the migration and redistribution of the photoreceptor cells, occurs *ex utero* in premature infants^7^.

Spectral-domain optical coherence tomography (SD-OCT) studies have demonstrated several microstructural abnormalities in the eyes of premature infants, such as retention of the inner retinal layers (IRL) at the fovea and an absence of foveal depression in individuals aged 2–18 years with a history of premature birth^8–12^.

Laser therapy is considered to be the most effective treatment for ROP^2,4^. However, laser therapy destroys the peripheral retina and retards abnormal growth of peripheral blood vessels^2^. There is no consensus regarding the effect of laser treatment on retinal immaturity; however, the signs of immaturity could be a predictor of visual function.

Therefore, this study aimed to determine whether laser photocoagulation affects retinal maturation. To accomplish this, we analyzed the foveal morphology in children treated with laser photocoagulation for ROP. The findings in these children were compared to those in children whose ROP spontaneously regressed and age-matched full-term children.

## Results

Seventy-four children with a history of ROP were divided into two groups: the laser-treated ROP group and the spontaneously regressed ROP group. The laser-treated ROP group consisted of 41 patients, including 20 boys and 21girls, and the mean age was 5.0 ± 0.8 years (mean ± standard deviation). The spontaneously regressed ROP group consisted of 33 patients (18 boys and 15 girls) with a mean age of 4.9 ± 0.8 years. The control group consisted of 30 children (12 boys and 18 girls) with a mean age of 5.0 ± 1.4 years (Table1).

**Table 1:** Demographics of the patients and controls.

	Treated ROP (n = 41)	Regressed ROP (n = 33)	Control (n = 30)	P value^a^ t-ROP vs r-ROP vs control	*P* value^b^ t-ROP vs r-ROP	*P* value^b^ t-ROP vs control	*P* value^b^ r-ROP vs control
Gestational age [week]	26.1 (2.3)	28.8 (3.3)	39.0 (1.9)	0.0001	0.0001	0.0001	0.0001
Birth weight [g]	856.0 (283.0)	1094.3 (472.2)	2988.7 (462.1)	0.0001	0.0001	0.0001	0.0001
Sex [Boy/Girl]	20/21	18/15	12/18	0.926			
Assessment age [years]	5.0 (0.8)	4.9 (0.8)	5.0 (1.4)	0.812			
Visual acuity [logMAR]	0.04 (0.13)	0.06 (0.18)	− 0.04 (0.05)	0.009	0.57	0.063	0.008
Spherical equivalent [D]	− 1.50 (3.00)	+ 1.00 (1.75)	+ 0.73 (1.41)	0.090			
Axial length [mm]	21.7 (1.3)	21.4 (0.6)	22.2 (0.9)	0.006	0.219	0.179	0.004
ROP stage	3.0 (0)	1.2 (1.1)		0.0001	0.0001		

Data are expressed as mean (standard deviation).

*P* value: ^a^analysis of variance (ANOVA) ^b^Tukey.

logMAR: logarithm of the minimum angle of resolution, ROP: retinopathy of prematurity, t-ROP: treated ROP, r-ROP: regressed ROP.

The age and sex distributions were not significantly different between the patients with ROP and the control group (analysis of variance, ANOVA).

The gestational age and birth weight were significantly lower in the laser-treated ROP group than that in the spontaneously regressed ROP and control groups (*P* = 0.0001, ANOVA).

The mean better best-corrected visual acuity (BCVA) at the initial examination was 0.04 ± 0.13, 0.06 ± 0.18, and − 0.04 ± 0.05 logarithm of the minimum angle of resolution (logMAR) units in the laser-treated, regressed ROP, and control groups, respectively. The BCVA was significantly better in the control group (*P* = 0.009, ANOVA). All the patients had a BCVA of 20/40 or better.

The mean refractive error was − 1.50 ± 3.00, + 1.00 ± 1.75, and + 0.73 ± 1.41 diopter (D) in the laser-treated, regressed, and D control eyes, respectively (*P* = 0.090, ANOVA). The mean axial length was 21.7 ± 1.3, 21.4 ± 0.6, and 22.2 ± 0.9 mm in the treated, regressed, and control eyes, respectively (*P* = 0.006, ANOVA; Table 1). In the laser-treated ROP group, ROP was treated at stage 3 in all the eyes. In the regressed ROP group, ROP was spontaneously regressed at stage 0, 1, 2, and 3 in 10, 9, 5, and 7 eyes, respectively.

### Foveal thickness

The mean foveal thickness was 264.0 ± 26.0, 241.1 ± 22.9, and 214.4 ± 13.4 µm in the laser-treated, spontaneously regressed, and control eyes, respectively (*P* = 0.0001, ANOVA; Table 2). The fovea was significantly thicker in the laser-treated eyes compared to the regressed and control eyes. The inter-observer reproducibility was excellent (intraclass correlation coefficients = 0.91).

**Table 2:** Optical coherence tomography findings of the patients and controls.

	Treated ROP (n = 41)	Regressed ROP (n = 33)	Control (n = 30)	*P* value^a^ t-ROP vs r-ROP vs. control	*P* value^b^ t-ROP vs r- ROP	*P* value^b^ t-ROP vs. control	*P* value^b^ r-ROP vs. control
Foveal thickness [µm]	264.0 (26.0)	241.1 (22.9)	214.4 (13.4)	0.0001	0.0001	0.0001	0.0001
Foveal depression [µm]	59.0 (31.0)	75.3 (34.7)	124.5 (17.1)	0.0001	0.001	0.0001	0.0001
ONL Thickness [µm]	120.8 (12.1)	110.5 (20.0)	84.3 (12.4)	0.0001	0.0001	0.0001	0.0001
IS Thickness [µm]	46.5 (5.5)	43.2 (5.6)	38.5 (8.8)	0.001	0.90	0.0001	0.0001
OS Thickness [µm]	41.3 (4.0)	40.9 (5.0)	43.9 (3.2)	0.01	0.90	0.02	0.03
IRL Thickness [µm]	11.8 (9.0)	3.7 (11.2)	0 (0)	0.0001	0.12	0.0001	0.019
				*P* value^c^			
Detection rates of inner retinal layer [%]	51.2	12.0	0	0.0001			

Data are expressed as mean (standard deviation).

*P* value : ^a^Analysis of variance (ANOVA) ^b^Tukey ^c^χ^2^-test.

logMAR: logarithm of the minimum angle of resolution, ROP: retinopathy of prematurity, ONL: outer nuclear layer, IS: inner segment, OS: outer segment, IRL: inner retinal layer, t-ROP: treated ROP, r-ROP: regressed ROP.

The mean foveal depression was 59.0 ± 31.0, 75.3 ± 34.7, and 124.5 ± 17.1 µm in the treated, regressed, and control eyes, respectively. The foveal depression was significantly shallower in the laser-treated eyes than that in the regressed and control eyes (*P* = 0.0001, ANOVA; Table 2). The mean thickness of the outer nuclear layer (ONL) was significantly thicker in the treated eyes than that in the regressed eyes and control eyes (*P* = 0.0001, ANOVA; Table 2). The length of the inner segment (IS) was significantly thicker in the treated and regressed ROP eyes than that in the control eyes (*P* = 0.0001, ANOVA; Table 2). IRL was significantly thicker in the laser-treated eyes than that in the regressed eyes and control eyes (*P* = 0.0001, ANOVA; Table 2). IRL was detected in 51.2% of the treated eyes and 12.0% of the regressed eyes; thus, IRL was significantly preserved in the laser-treated eyes, whereas it was not detected in the control eyes (*P* = 0.0001, χ^2^-test; Table 2). The resistance of IRL was attributed to a delay in foveal maturation^9^. In the analysis of covariance (ANCOVA) adjusted with the gestational age and ROP, the foveal, IS, and IRL thickness was thicker in the laser treated group than regressed ROP and control group (Table 3).

**Table 3:** Effect of the retinal layer thickness to the retinopathy of prematurity and the laser treatment.

	ROP (Treated ROP + Regressed ROP) (n = 74)	No ROP (Control) (n = 30)	*P*^a^	Laser treatment (Treated ROP) (n = 41)	No Laser treatment (Regressed ROP + Control) (n = 63)	*P*^b^
Foveal thickness [µm]	242.0	243.3	0.90	252.0	236.1	0.005
Difference(95%CI)	234.5 to 249.5	227.2 to 259.5		244.3 to 260.0	230.1 to 242.0	
Foveal depression [µm]	78.2	95.0	0.26	78.1	86.3	0.27
Difference(95%CI)	68.1 to 88.3	73.3 to 113.7		67.6 to 88.6	78.3 to 94.3	
ONL thickness [µm]	107.9	104.7	0.66	109.8	105.2	0.22
Difference(95%CI)	102.8 to 113.0	93.8 to 115.6		104.5 to 111.1	101.1 to 109.2	
IS Thickness [µm]	45.1	38.2	0.05	45.4	41.6	0.03
Difference(95%CI)	42.7 to 47.5	33.1 to 43.3		43.0 to 47.9	39.7 to 43.4	
OS Thickness [µm]	42.7	40.0	0.19	43.1	41.2	0.08
Difference(95%CI)	41.3 to 44.1	37.0 to 43.0		41.6 to 44.1	40.1 to 42.3	
IRL Thickness [µm]	8.6	0	0.01	13.4	4.8	0.01
Difference(95%CI)	5.9 to 11.3			8.9 to 17.9	0.9 to 8.7	

Data are expressed as adjusted mean based on analysis of covariance (ANCOVA).

*P* value ^a^ANCOVA adjusted for gestational age and laser treatment ^b^ANCOVA adjusted for gestational age and ROP.

ROP: retinopathy of prematurity, CI, confidence interval, ONL: outer nuclear layer, IS: inner segment, OS: outer segment, IRL: inner retinal layer.

In the univariate analysis, better BCVA was positively associated with mature gestational age, larger birth weight, smaller refractive error, deeper foveal depression, and thinner IS thickness in ROP treated eyes (Pearson’s coefficient; Table 4). In the regressed and control eyes, BCVA was not associated with the foveal morphology (Pearson’s coefficient; Table 4). In the multiple linear regression analysis of ROP and controls, better BCVA was significantly associated with deeper foveal depression (Standardized β = − 0.278, *P* = 0.039; Table 5). In the multiple linear regression analysis of the ROP and control eyes, deeper foveal depression was significantly associated with mature gestational age (Standardized β = 0.733, *P* = 0.008; Table 6).

**Table 4:** Effect of the clinical elements on the best corrected visual acuity (logMAR).

	Treated ROP (n = 41)		Regressed ROP (n = 33)		Control (n = 30)	
	r	*P* value	r	*P* value	R	*P* value
Gestational age (week)	− 0.347	0.026	− 0.086	0.634	0.032	0.946
Birth weight (g)	− 0.335	0.032	− 0.143	0.426	0.018	0.963
Spherical equivalent (D)	− 0.459	0.003	− 0.452	0.008	0.266	0.155
Axial length (mm)	0.287	0.095	0.231	0.196	0.01	0.958
Foveal thickness (µm)	− 0.018	0.912	− 0.027	0.881	− 0.158	0.404
Foveal depression (µm)	− 0.394	0.01	− 0.214	0.231	− 0.04	0.832
ONL Thickness (µm)	− 0.012	0.938	0.225	0.207	− 0.01	0.958
IS Thickness (µm)	− 0.260	0.1	0.068	0.739	− 0.081	0.699
OS Thickness (µm)	0.004	0.980	0.004	0.985	− 0.242	0.197
IRL Thickness(µm)	0.063	0.609	0.013	0.942		
ROP Stage	0.075	0.63	0.088	0.628		
Frequency of laser treatment	0.083	0.183				

Univariate linear regression analysis was performed.

logMAR: logarithm of the minimum angle of resolution, ROP: retinopathy of prematurity, ONL: outer nuclear layer, IS: inner segment, OS: outer segment, IRL: inner retinal layer.

**Table 5:** Multiple linear regression analysis between visual acuity and independent variables.

Independent variable	Visual acuity (logMAR)	
	Standardized β	*P* value
Gestational age (week)	0.063	0.865
Birth weight (g)	− 0.399	0.253
Foveal depression (µm)	− 0.278	0.039
IS thickness (µm)	− 0.065	0.539
ROP stage	− 0.133	0.562
Laser treatment	− 0.136	0.421

logMAR: logarithm of the minimum angle of resolution, ROP: retinopathy of prematurity, IS: inner segment.

**Table 6:** Multiple linear regression analysis between foveal depression and independent variables.

Independent variable	Foveal depression (µm)	
	Standardized β	*P* value
Gestational age (week)	0.733	0.008
Birth weight (g)	− 0.187	0.470
Axial length (mm)	− 0.088	0.228
ROP stage	− 0.258	0.117
Laser treatment	0.026	0.829

ROP: retinopathy of prematurity.

### Representative ROP patient

**Figure 1:**
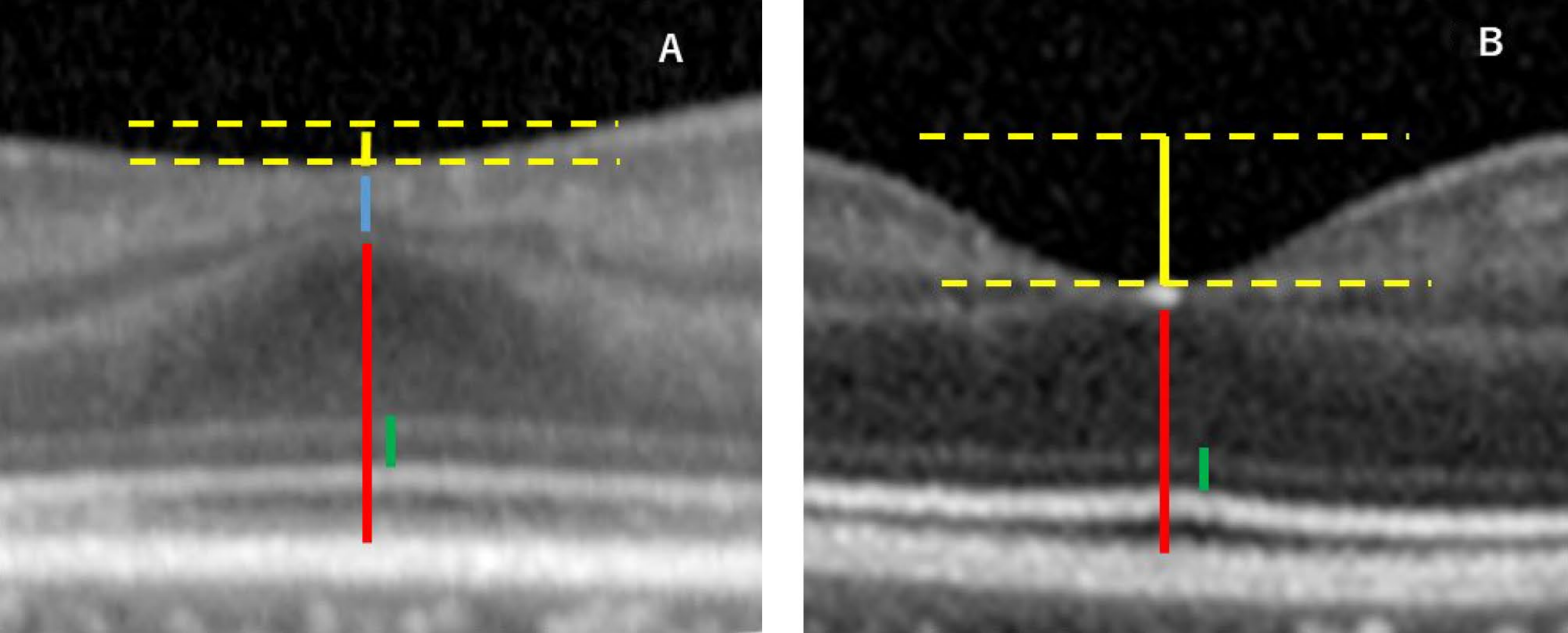
(**A**) Optical coherence tomography (OCT) images of a representative patient with retinopathy of prematurity (ROP) who underwent laser treatment. The foveal depression is shallow, at 22.5 µm in the OCT images, and IRL of the fovea is preserved (Fig. 1A). The inner retinal layer (blue line) at the fovea is still present. The foveal thickness (inner retinal layer and outer retinal layer, i.e., blue and red line, respectively) is 239 µm, the IS and OS lengths are 50 and 40 µm, respectively. Yellow line: foveal depression; blue line: inner retinal layer; red line: outer retinal layer; green line: IS length. (**B**) OCT image of a representative eye from the spontaneously regressed ROP group. The foveal depression is 108.5 µm in the OCT images (Fig. 1B). IRL of the fovea is not detected and the foveal thickness (red line, outer retinal layer) is 237 µm. IS length is 44 µm and OS length is 43 µm. Yellow line: foveal depression, red line: outer retinal layer, green line: IS length.

Case 1: A 6-year-old boy was born at 25 weeks gestation and weighed 690 g at birth. He was classified with Zone 1 and stage 3 ROP with plus disease. The patient was treated with laser photocoagulation at 33-weeks-of-age. His BCVA was 20/40 Snellen equivalent in both eyes. The foveal depression was shallow at 22.5 µm in the OCT images, and the IRL of the fovea was preserved (Fig. 1A). The fovea was 239 µm thick. The ONL was 133 µm, IS was 50 µm, Outer segment (OS) was 40 µm, and IRL was 33 µm.

Case 2: A 4-year-old girl was born at 24 weeks gestation and weighed 640 g at birth. She was classified as Zone 3 stage 3 ROP without plus disease. ROP spontaneously regressed after 44 weeks in this case. Her BCVA was 20/25 Snellen equivalent in both eyes. The foveal depression measured 108.5 µm in the OCT images (Fig. 1B). IRL of the fovea was not detected and the foveal thickness was 218 µm. The ONL was 92 µm, IS was 44 µm, OS was 43 µm, and IRL was not detected.

The foveal depression was deeper, IRL was thinner, and the visual acuity was better in the second case than that in the first treated case.

## Discussion

We found that the patients with a history of ROP demonstrated macular morphological differences, such as, retention of IRL, shallower foveal depression, and thicker ONL and IS, compared to full-term control children. These findings suggest an alteration in the retinal developmental process in eyes with ROP.

### OCT findings

We observed a thicker ONL and resistance of IRL in children with ROP, which is in agreement with the results of previous reports^9,13,14^. Preterm birth between 24 and 28 weeks of gestation has been proposed to be a critical period associated with failure of normal migration of IRL away from the fovea, resulting in increased foveal thickness and resistance of IRL^13,14^. The migration of IRL appears to be independent of the outer retinal development, as previously suggested^15^. This disparity in maturation could be associated with the variation in the vascular supply between the inner and outer retina. The vasculature of the outer retina develops during the late stage of gestation^16^. IRL matures completely at the time of full-term birth; however, maturity of the outer retina is not attained until a few years after birth^13,14^. The maturation of the outer retina includes the development of the IS and OS layers, which occurs after birth and continues up to 5 years of age^7,17^. These processes are disrupted by preterm birth and ROP. Thicker ONL and IS in children with ROP could be attributed to a delay in foveal maturation^18^.

Visual acuity.

BCVA of the treated and regressed ROP patients was worse than that of the control children, although all the patients with ROP had 20/40 or better BCVA. In previous reports, some studies found that a thicker fovea resulted in poorer visual acuity in childhood^10,19^. Another study found no relationship between foveal thickness and visual acuity^20^.There is no consensus regarding the relationship between visual acuity and infant prematurity. In our study, BCVA of the treated ROP patients was significantly associated with early preterm birth, light birth weight, large refractive error,, thick IS, and shallow foveal depression. Early preterm birth and light birth weight are signs of infant prematurity. Large refractive error in patients with ROP is affected by laser treatment^10,19^. Shallow foveal depression and thicker IS in patients with ROP were signs of immaturity of the foveal development^18^. In our study, thicker IS and foveal thickness was significantly associated with the laser treatment. Thus, BCVA is related to infant prematurity, foveal prematurity, and laser treatment.

### The laser treatment of ROP

IS in the eyes of patients with ROP was thicker than that in the control eyes. Premature neonates had a thin photoreceptor layer at the fovea on the SD-OCT images^7^. These studies demonstrated the presence of significant foveal cone specialization in the absence of a foveal pit^21^. The migration of the cells in IRL was independent of the photoreceptor development in the outer retinal layer^15^. The results of a recent study showed that maturation of the outer retinal layer and photoreceptors is delayed in laser-treated ROP eyes^22^. Vogel et al. reported that infants treated with intravitreal injection of bevacizumab (IVB) for ROP had more rapid outer retinal thickening at the fovea, while those treated with laser therapy showed delayed development of the ellipsoid zone at the fovea^22^. However, the structure–function correlations in these regions remain poorly understood. We studied children aged 4–6 years when the retina is still developing^16,23^. In these ages, foveal development can last, and the outer retina of patients with ROP is still developing^23^. Celik G et al. reported that at 5 years of age, foveal thickness was significantly thicker in laser treated ROP eyes than in IVB treated ROP eyes^24^. In this report, foveal morphology was not discussed. Our results suggest that infant prematurity and laser treatment affect foveal morphology. Deeper foveal depression was associated with mature gestational age and laser treatment, which affected the foveal, IRL, and IS thickness.

### Limitations

This study had certain limitations. The number of patients with ROP and controls was small. In addition, all the participants were Japanese. Considering previous studies, Hispanic neonates reportedly have higher rates of severe ROP compared with non-Hispanic neonates, while non-Hispanic Black neonates reportedly have lower rates of ROP than non-Hispanic White neonates^25,26^.

We manually measured the retinal layers owing to segmentation errors during the use of automated software^27^. The retinal thickness was measured using Spectralis OCT and Cirrus OCT. The differences between the two SD-OCT systems were owing to the differences in their built-in software algorithms^28^; we measured the retinal thickness using ImageJ software. We did not have an additional IVB group.

## Conclusion

In conclusion, the morphology of the fovea in the eyes of children with laser-treated ROP was significantly different from that of spontaneously regressed ROP and control eyes. In the patients with ROP and controls, better BCVA was associated with deeper foveal depression, which was associated with mature gestational age. Our results suggest that infant prematurity and laser treatment affect foveal morphology and visual acuity.

## Methods

This multicenter retrospective study included patients with a history of ROP who were diagnosed and followed up at the Nara Medical University Hospital, Tokushima University Hospital, Hyogo Medical University Hospital, Kobe University Hospital, Sapporo City General Hospital, Shiga Medical University Hospital, and Kurume University Hospital between April 2012 and December 2019.

The protocol for this study conformed to the tenets of the Declaration of Helsinki and was approved by the Internal Review Boards of the Nara Medical University, Tokushima University, Hyogo Medical University, Kobe University Hospital, Sapporo City General Hospital, Shiga Medical University Hospital, and Kurume University Hospital. For research involving human participants who were minors, informed consent was obtained from their parents for permitting performing measurements and reviewing their medical records.

All the patients had a history of ROP and were followed up for 4–6 years. Patients who were capable of cooperating with the OCT examinations were included. Patients with organic eye diseases, history of intraocular surgery, cataracts, glaucoma, or any retinal disorders were excluded. During acute phase ROP, ROP was examined at least at one visit per week. Laser-treated ROP patients were treated at the pre-threshold ROP or threshold ROP stage^29^. Laser treatment was performed in all the quadrants in the laser treated eyes. The patients with spontaneously regressed ROP presented with stage 3 or less, and none of them had received any treatment for ROP previously.

Age-matched, full-term children with normal ocular findings were recruited from the Nara Medical University Hospital and the nursery school of the Tokushima University Hospital for the control group. In control group, children with myopia greater than -1.00 D or with hyperopia greater than + 2.00 D were excluded.

All the patients and controls were examined using slit-lamp biomicroscopy, extraocular motility assessment, subjective cycloplegic refractions (1% cyclopentolate and 2.5% phenylephrine), dilated ophthalmoscopy, and SD-OCT imaging. The same examination procedures were used for both the eyes of the patients with ROP and the right eyes of the controls. One severe eye per patient was selected for the analysis.

The retinal thickness, axial length, and BCVA of 18 eyes of the control patients have been reported^30–32^. The visual acuity was measured using a standard Snellen chart at 5 months and was converted to the logMAR units for statistical analyses. The axial length was measured using IOL Master (Carl Zeiss Meditec, Jena, Germany) and AL-2000 (TOMEY, Nagoya, Japan). The refractive error (spherical equivalent), gestational age, birth weight, sex, and history of laser treatment were also evaluated.

### Measurements of retinal layer thickness

The retinal layer thickness was determined using SD-OCT (Spectralis, Heidelberg Engineering, Heidelberg, Germany; Cirrus SD-OCT, Carl Zeiss Meditec, Jena, Germany and RS-3000, Nidek, Gamagori, Japan). The thicknesses of the fovea, IRL, ONL, and photoreceptor IS and OS layers were measured in the temporally scanned OCT images of the fovea. The thickness measurements at 1 mm nasal and temporal to the fovea were also measured as the parafoveal thickness. The foveal depression was calculated by subtracting the central foveal thickness from the mean parafoveal thickness.

The thickness of each retinal layer was determined by two experienced retinal specialists who were blinded to the diagnosis. They evaluated the retinal thickness independently and manually using an open-access software ImageJ (version 1.50a; NIH, Bethesda, Maryland, USA). The ONL thickness was measured as the distance from the outer border of the inner limiting membrane (ILM) to the external limiting membrane (ELM). The IS length was measured as the distance between the ELM and outer border of the ellipsoid zone (EZ). The OS length was measured as the distance between the outer border of the EZ and the inner border of the retinal pigment epithelium. The arithmetic means of the two examiners were used for the statistical analyses. The inter-observer reproducibility was evaluated using intraclass correlation coefficients.

### Statistical analyses

The data are expressed as means ± standard deviation. The gestational birth date, age at the time of the examination, BCVA, axial length, refractive error (spherical equivalent), and foveal structures of the ROP and control eyes were compared using one-way ANOVA with post-hoc Tukey tests and ANCOVA adjusted with the gestational age. The presence of the IRL was compared using theχ^2^-test. Univariate and multivariate linear regression analyses were performed to determine the significance of the correlations between the BCVA and OCT findings of ROP and control eyes. The potential confounders (gestational age, birth weight, axial length, laser treatment, and ROP stage) were included in the multivariate linear regression analyses. The standardized coefficients (ß) were calculated for each independent variable. Statistical significance was set at *p* < 0.05. Statistical analyses were performed using licensed statistical software (SPSS version 22.0; SPSS Inc., Chicago, IL, USA).

## Data Availability

The data that support the findings of this study are available from the corresponding author, T.N., upon reasonable request.
